# Supplementary material for: A global lipid map defines a network essential for Zika virus replication
Source: Nat Commun. 2020 Jul 21;11:3652. doi: 10.1038/s41467-020-17433-9 (PMC7374707; doi:10.1038/s41467-020-17433-9)
Supplement: Supplementary file 1 — Supplementary Information [file 41467_2020_17433_MOESM1_ESM.pdf]

Supplementary Information

A global lipid map defines a network essential for Zika virus Replication

Hans C. Leier[1], Jules B. Weinstein[1], Jennifer E. Kyle[2], Joon-Yong Lee[2], Lisa M. Bramer[2], Kelly G. Stratton[3], Douglas Kempthorne[1,4], Aaron R. Navratil[5], Endale G. Tafesse[6], Thorsten Hornemann[7], William B. Messer[1,8], Edward A. Dennis[5], Thomas O. Metz[2], Eric Barklis[1], Fikadu G. Tafesse[1]

[1]Department of Molecular Microbiology & Immunology, Oregon Health & Science University (OHSU), Portland, OR 97239, USA

[2]Biological Sciences Division, Earth and Biological Sciences Directorate, Pacific Northwest National Laboratory (PNNL), Richland, WA 99352, USA

[3]Computing and Analytics Division, National Security Directorate, PNNL, Richland, WA 99352, USA

[4]Center for Diversity and Inclusion, OHSU, Portland, OR 97239, USA.

[5]Departments of Chemistry & Biochemistry and Pharmacology, University of California San Diego School of Medicine, La Jolla, CA 92093, USA

[6]Department of Plant Sciences, University of Saskatchewan, Saskatoon SK S7N 5A8, Canada

[7]Zurich Center for Integrative Human Physiology (ZIHP), University of Zurich, Switzerland

[8]Department of Medicine, Division of Infectious Diseases, OHSU, Portland, Oregon 97239, USA

Correspondence and requests for materials should be addressed to F.G.T. (email: tafesse@ohsu.edu)

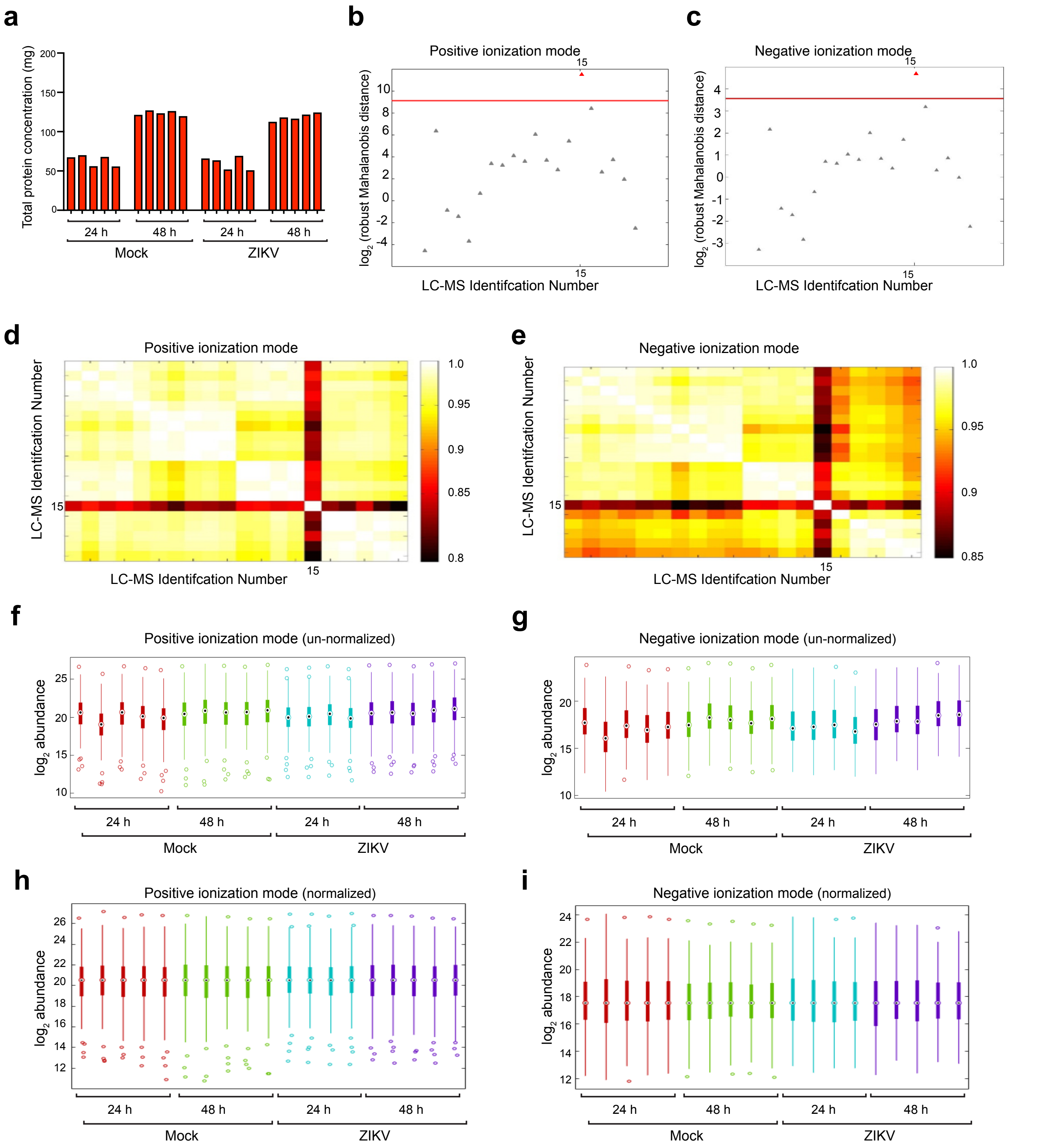

Supplementary Fig. 1 Quality control and pre-processing of lipidomic data sets. **a** Protein content of cell lysates used for lipidomics experiments. **b, c** The robust Mahalanobis distance (RMD) analysis from instrument runs in positive and negative ionization mode. RMD-PAV identifies positive- and negative-mode LC-MS datasets that are extreme deviants from the remaining datasets (infected, 24 hpi; sample #15). **d, e** Heatmap of Pearson correlations confirms the presence of a single outlier sample (infected, 24 hpi; sample #15). Sample #15 was excluded from further analysis. **f-i** Log_2 abundance of lipids across the full mass spectrometry dataset before (**f, g**) and after (**h, i**) normalization. Boxplots represent median (highlighted point) and 25th to 75th percentile of data (lower and upper bounds of boxes, respectively); whiskers extend to maximum and minimum samples within 1.5 times the interquartile range. *n* = 5 biologically independent samples for each condition. See also Supplementary Data 1.

a

b

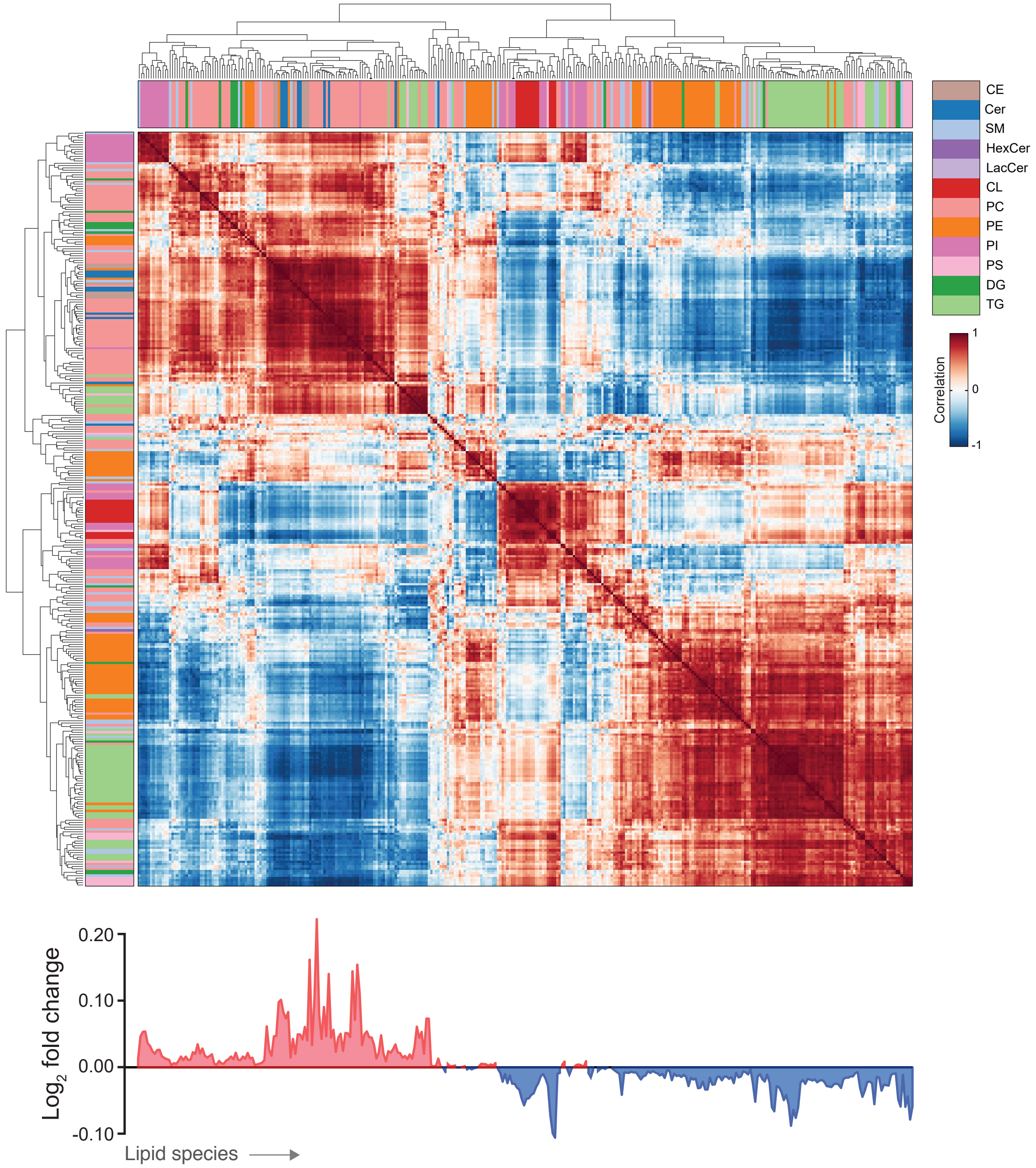

Supplementary Fig. 2 Map of lipid correlations during ZIKV infection. **a** Correlation matrix of the 340 lipid species at 48 hpi. Each cell represents the correlation of two lipid species across the five mock and five infected sample collected 48 hpi, with direction and strength of correlation indicated by heatmap. Colored barcodes identify the subclasses of lipid species, arranged by hierarchical clustering. **b** Log_2 fold change in abundance of lipid species at 48 hpi, corresponding to the matrix above shown in (**a**). See also Supplementary Data 1.

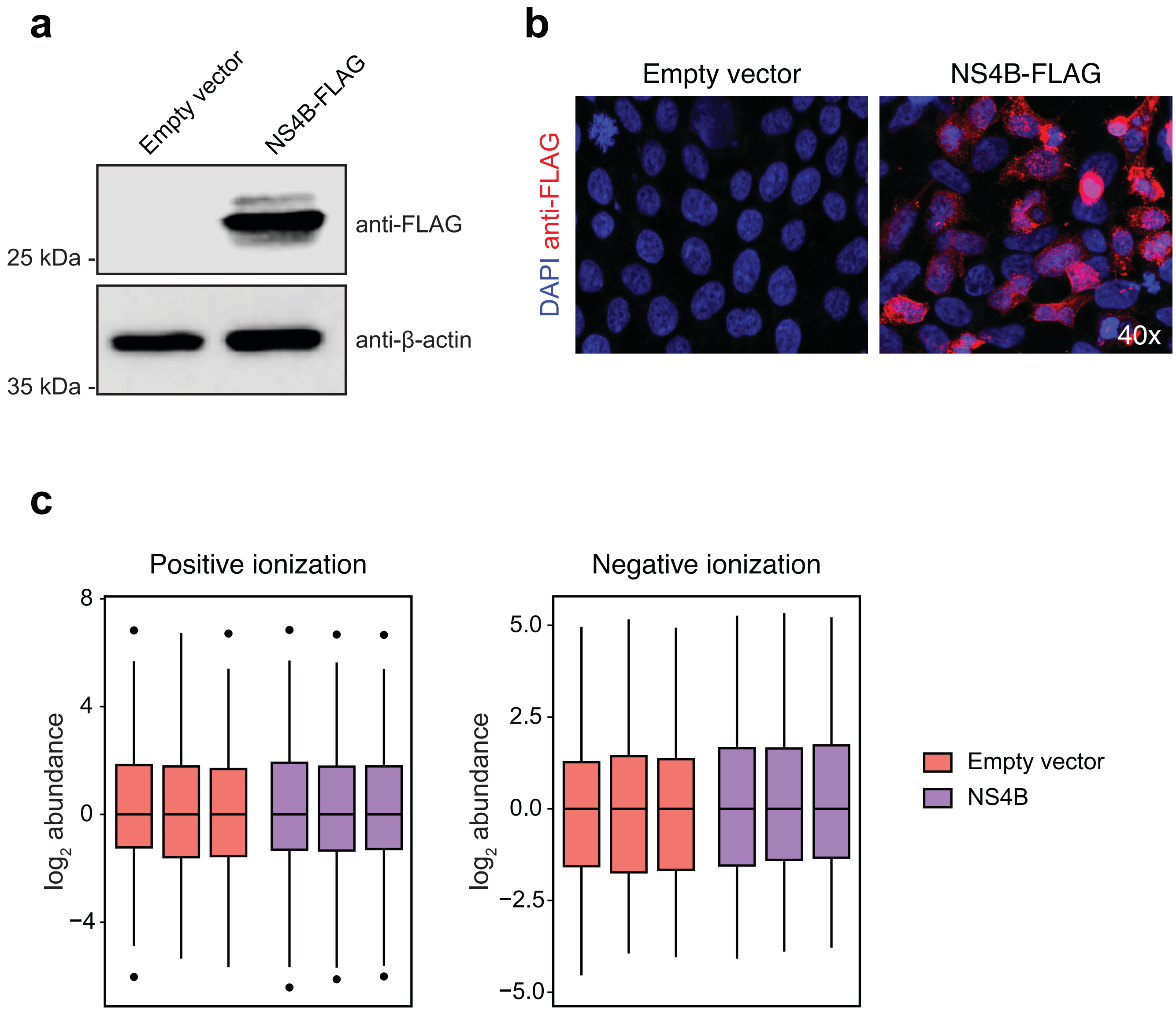

Supplementary Fig. 3 Confirmation of NS4B expression in HEK 293T cells and lipidomics quality control. **a, b** HEK 293T cells were transfected with empty vector or NS4B-FLAG expression vector. NS4B expression was examined with an anti-FLAG antibody by immunoblot (**a**) or confocal microscopy (**b**). **c** Log_2 abundance of lipids across the full mass spectrometry dataset after normalization. Boxplots represent median (highlighted point) and 25th to 75th percentile of data (lower and upper bounds of boxes, respectively); whiskers extend to maximum and minimum samples within 1.5 times the interquartile range. *n* = 3 independent biological replicates per condition. No outliers were identified. See also Supplementary Data 2.

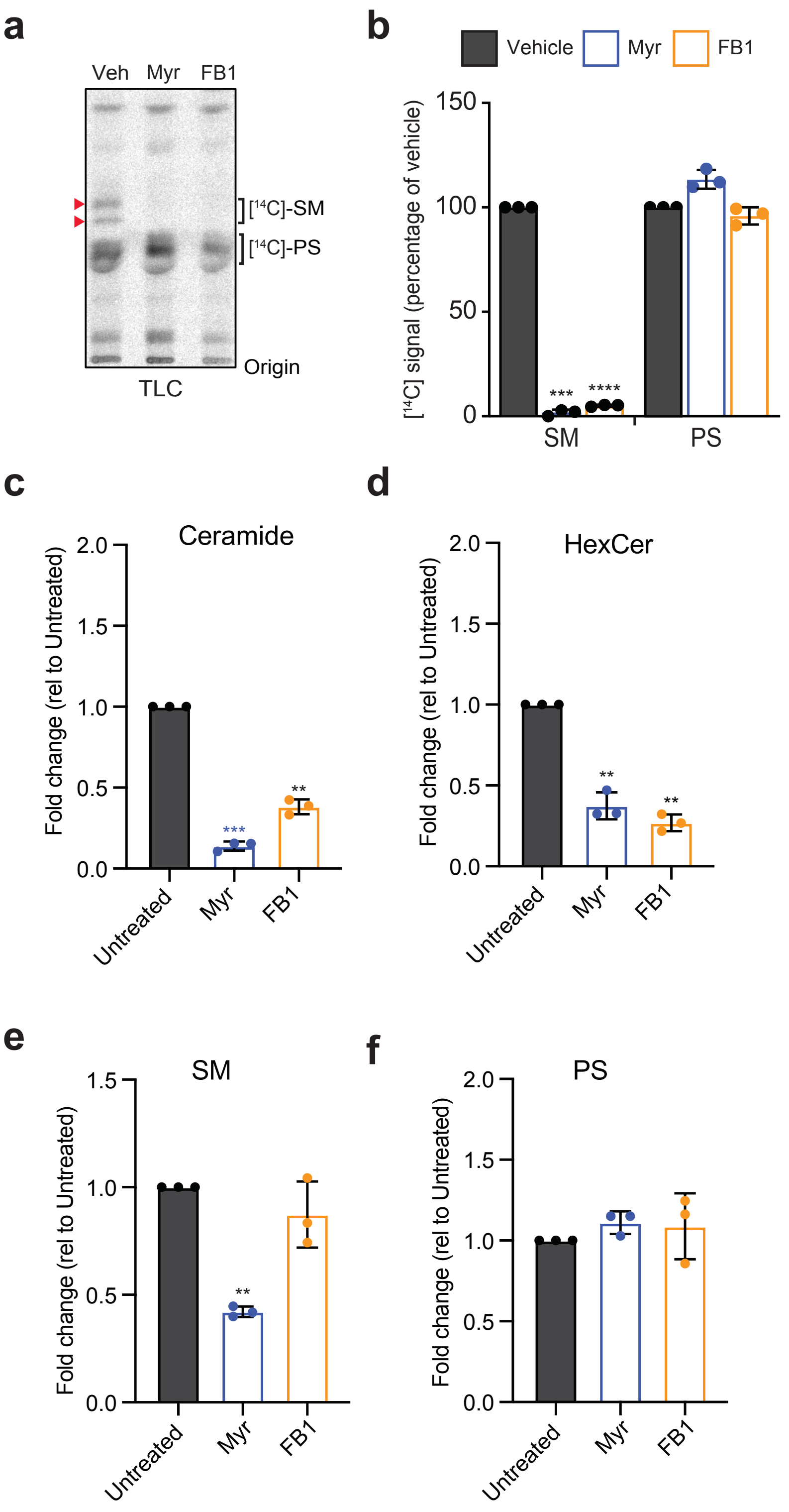

Supplementary Fig. 4 Treatment of Huh7 cells with myriocin or FB1 specifically blocks sphingolipid biosynthesis and depletes cellular sphingolipids. **a** Huh7 cells pretreated for three days with myriocin, FB1, or DMSO (vehicle) were labelled with the sphingolipid precursor 3-L-[^{14}C]-serine. Total cellular lipids were extracted and resolved by TLC and ^{14}C was visualized with autoradiography. TLC plate is representative of three independent experiments. Red arrows indicate SM bands. **b** [^{14}C]-SM and [^{14}C]-PS signals from (**a**) were quantified as percentage of the loading control. **c-f** Total lipids were extracted from control and inhibitor-treated cells and lipid profiling was performed using LC/MS. Levels of ceramide (**c**), Hexosylceramide (HexCer; **d**) sphingomyelin (SM; **e**) and phosphatidylserine (PS; **f**) are shown; n = 3 biological replicates). Data are mean ± SD. $^{**}P < 0.01$, $^{***}P < 0.001$, two-tailed Student's t-test.

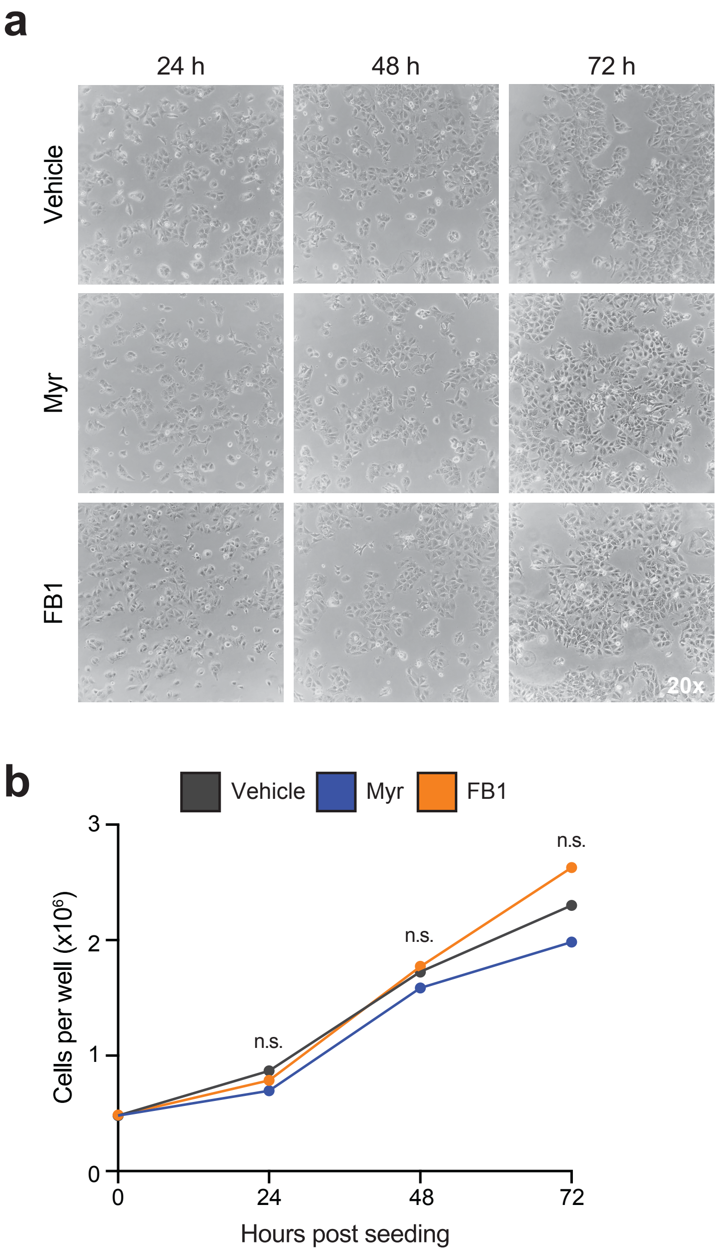

Supplementary Fig. 5 Treatment of Huh7 cells with myriocin or FB1 does not affect cell growth or morphology. **a** Representative 20x brightfield images of Huh7 cells treated for the indicated times with 30 μM myriocin, 5 μM FB1, or a vehicle control. **b** At 24, 48, and 72 hrs after seeding 50,000 cells/well in a 6-well plate, cells were trypsinized and counted with a hemocytometer. Data are mean ± SD. Representative images in (**a**) and quantitation in (**b**) are from *n* = 2 independent experiments, each performed in triplicate. n.s., not significant, unpaired two-tailed Student's t-test. See also the Source Data file.

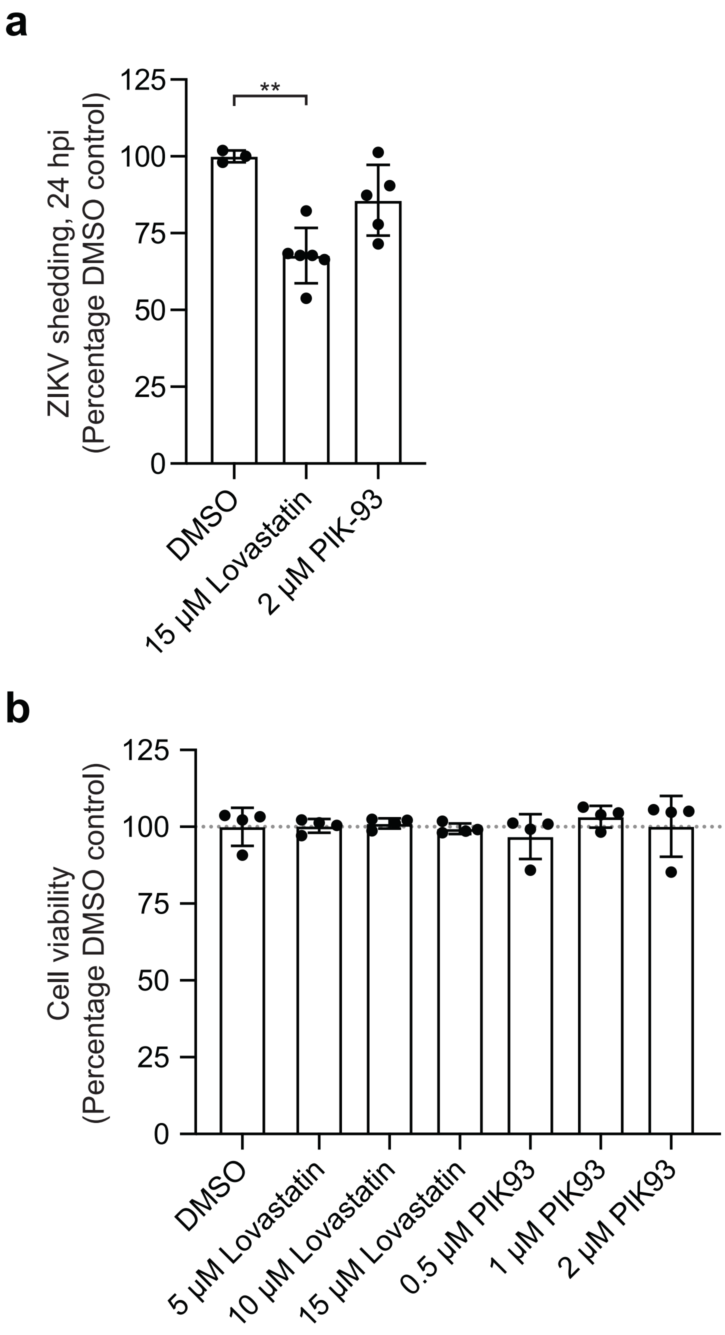

Supplementary Fig. 6 Effects of cholesterol and PI4P biosynthesis inhibition on ZIKV replication. **a** Huh7 cells were treated with a DMSO control or inhibitors of HMGR (lovastatin), or phosphatidylinositol 4-kinase IIIβ (PIK-93) at the indicated concentrations and infected with ZIKV at an MOI of 1. At 24 hpi, culture supernatants were collected and analyzed by plaque assay. Data are mean ± SD from $n = 3$ independent experiments; $^{**}P < 0.01$, one-way ANOVA with Tukey's multiple comparisons test. **b** Huh7 cells treated as in (**a**) with different concentrations of inhibitors were assessed for viability with a CellTiter Glo assay. n = 4 independent replicates. (**a, b**) Data are mean ± SD.

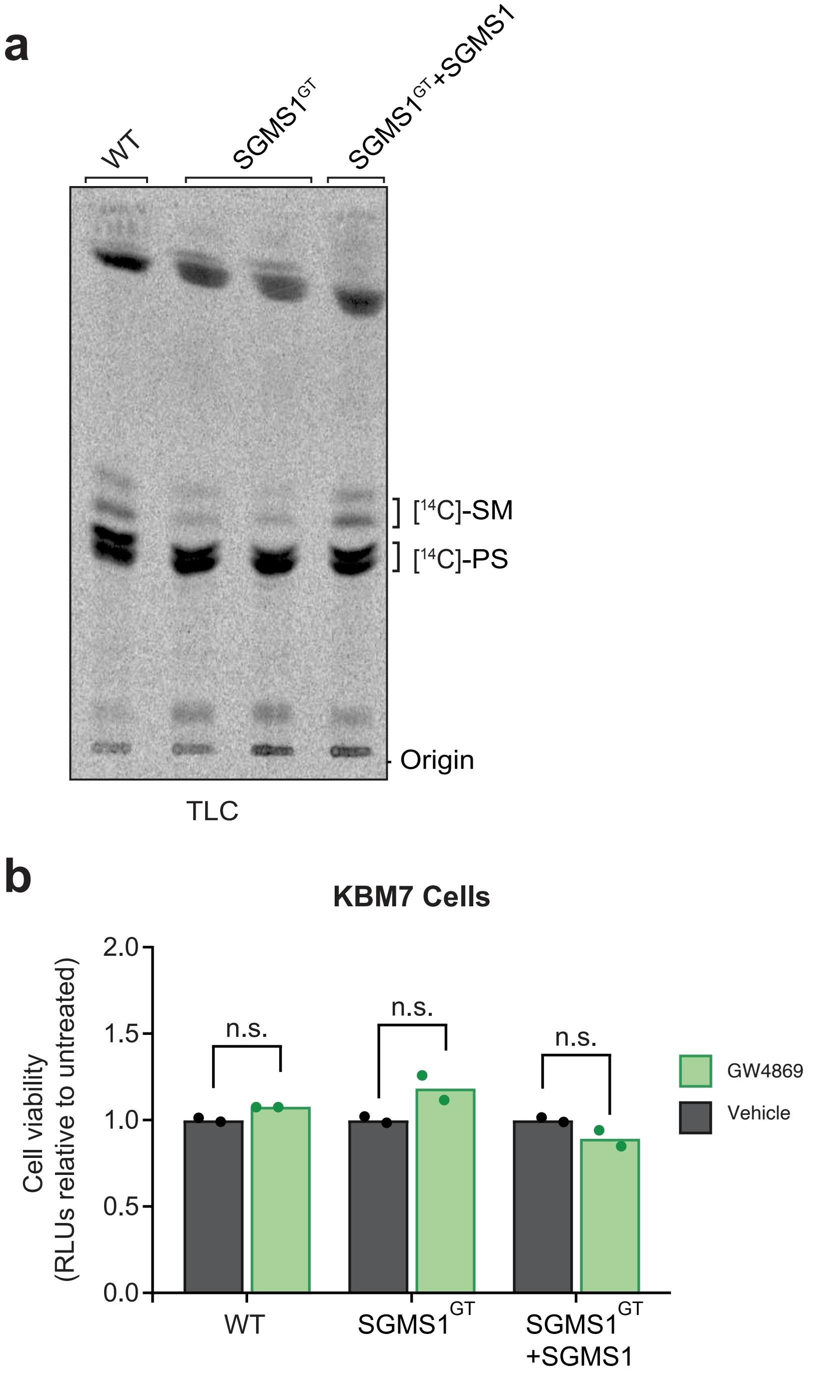

Supplementary Fig. 7 SGMS1^{GT} KBM7 cells produce reduced levels of SM, and treatment of these cells with GW4869 does not affect cell viability. **a** Wild type, SGMS1^{GT} and SGMS1^{GT}+SGMS1 cells were labelled with the sphingolipid precursor 3-L-[^{14}C]-serine. Total cellular lipids were extracted and analysed by TLC and autoradiography. TLC is representative of three independent experiments. **b** The three KBM7 cell lines were treated with 10 μM GW4869 for 24 hrs, then tested for viability compared to untreated cells by measuring ATP content (*n* = 2 independent experiments). Data are mean ± SD; n.s., not significant, two-tailed Student's t-test.

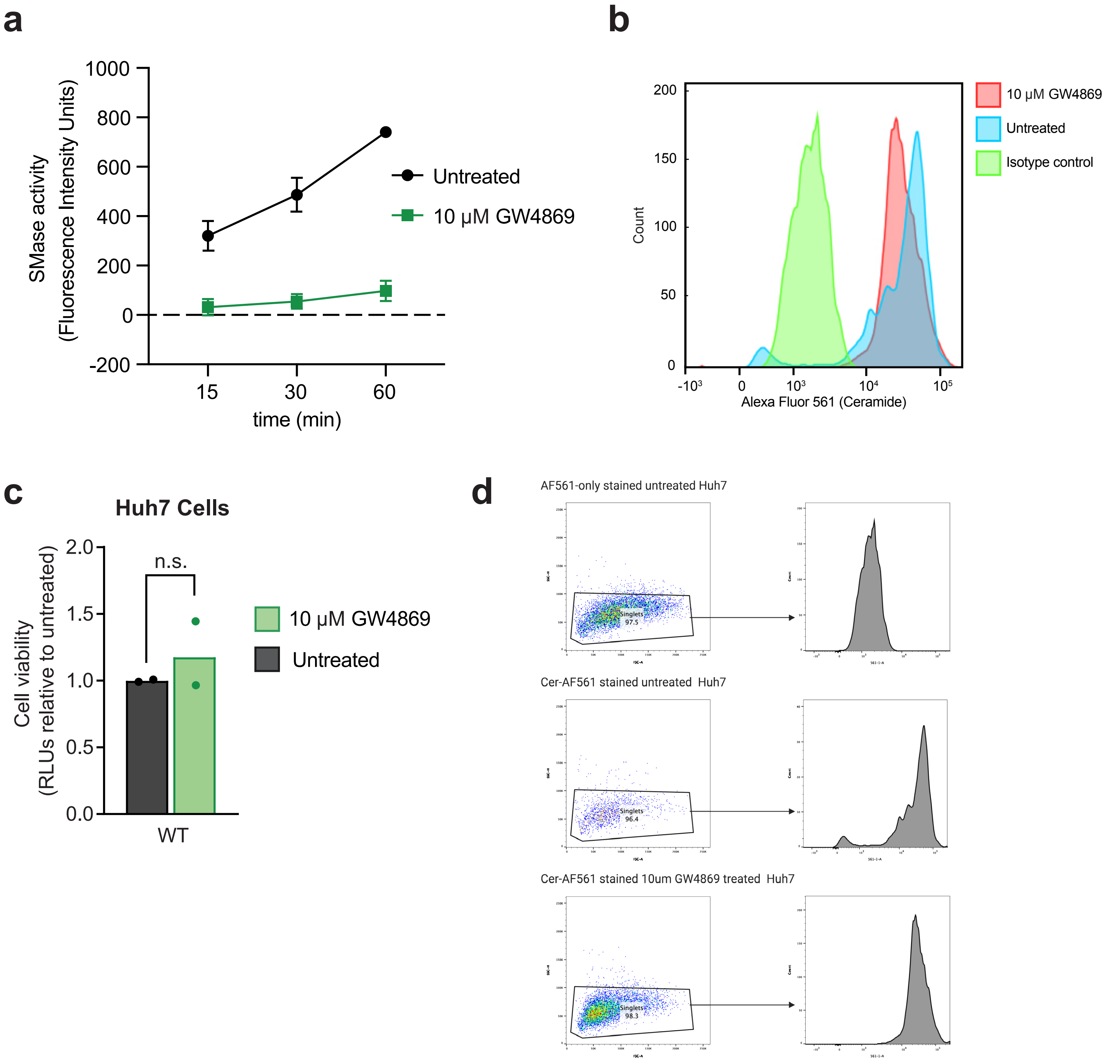

Supplementary Fig. 8 GW4869 inhibits cellular SMase activity. **a** Huh7 cells were treated with 10 μM GW4869 for 24 hrs, and the activity of cellular SMase was assayed using an Amplex Red sphingomyelinase kit (see Methods). **b** Huh7 cells were treated with GW4869 as in (**a**), stained with anti-ceramide monoclonal antibody, and analyzed by flow cytometry. **c** Huh7 cells were treated with 10 μM GW4869 as in (**a**), then tested for viability with the CellTiter Glo kit (n = 2 independent experiments). Data are mean ± SD; n.s., not significant, unpaired two-tail Student's *t*-test. **d** Gating scheme for data in (**b**). See also the Reporting Summary and Source Data file.

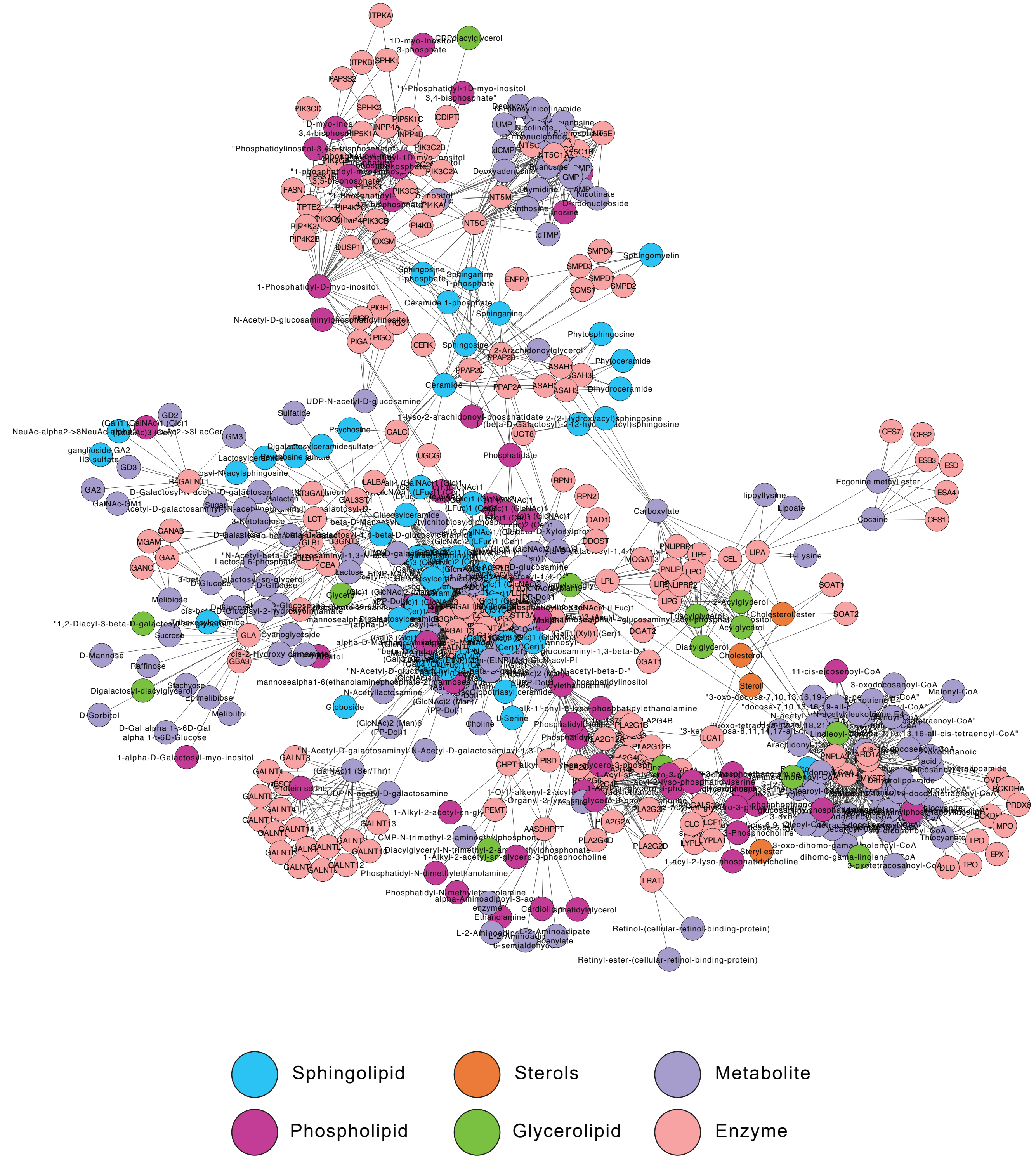

Supplementary Fig. 9 Map of lipid metabolic network. A model of the metabolic network producing the lipid subclasses identified through lipidomics was generated in Cytoscape using the MetScape 3 plugin. Lists of genes and compounds making up the network for each lipid subclass were accessed in MetScape 3 from the KEGG database.

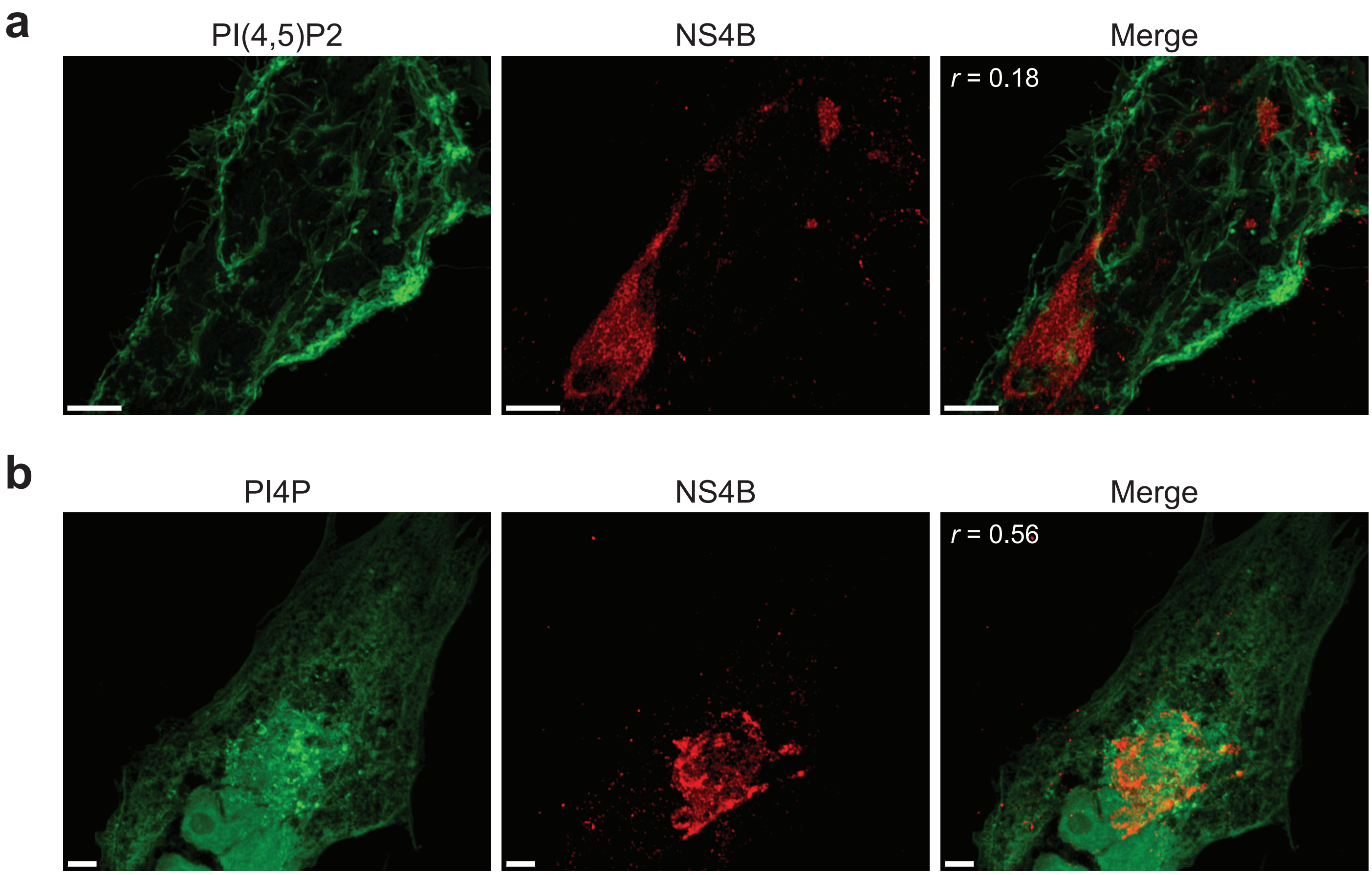

Supplementary Fig. 10 Distribution of PI lipids during ZIKV infection. **a, b** Huh7 cells were transfected with biosensors for PI(4,5)P2 (pLNCX-PH-PLCδ1-GFP) or PI4P (GFP-P4M-SidM) for 24 h, then infected with ZIKV (MOI = 10) for 24 h before fixation and staining with anti-NS4B. Colocalization between lipids and NS4B was assessed with Pearson's correlation coefficient, *r*. Scale bar, 5 μm. Images are representative of three independent experiments.
