# Supplementary material for: A global lipid map defines a network essential for Zika virus replication
Source: Nat Commun. 2020 Jul 21;11:3652. doi: 10.1038/s41467-020-17433-9 (PMC7374707; doi:10.1038/s41467-020-17433-9)
Supplement: Supplementary file 3 — Reporting Summary [file 41467_2020_17433_MOESM3_ESM.pdf]

nature research

Corresponding author(s): Fikadu G. Tafesse

Last updated by author(s): Jun 16, 2020

Reporting Summary

Nature Research wishes to improve the reproducibility of the work that we publish. This form provides structure for consistency and transparency in reporting. For further information on Nature Research policies, see our Editorial Policies and the Editorial Policy Checklist.

Statistics

For all statistical analyses, confirm that the following items are present in the figure legend, table legend, main text, or Methods section.

n/a	Confirmed	
☐	☒	The exact sample size (*n*) for each experimental group/condition, given as a discrete number and unit of measurement
☐	☒	A statement on whether measurements were taken from distinct samples or whether the same sample was measured repeatedly
☐	☒	The statistical test(s) used AND whether they are one- or two-sided<br>*Only common tests should be described solely by name; describe more complex techniques in the Methods section.*
☒	☐	A description of all covariates tested
☐	☒	A description of any assumptions or corrections, such as tests of normality and adjustment for multiple comparisons
☐	☒	A full description of the statistical parameters including central tendency (e.g. means) or other basic estimates (e.g. regression coefficient) AND variation (e.g. standard deviation) or associated estimates of uncertainty (e.g. confidence intervals)
☐	☒	For null hypothesis testing, the test statistic (e.g. *F*, *t*, *r*) with confidence intervals, effect sizes, degrees of freedom and *P* value noted<br>*Give P values as exact values whenever suitable.*
☒	☐	For Bayesian analysis, information on the choice of priors and Markov chain Monte Carlo settings
☐	☒	For hierarchical and complex designs, identification of the appropriate level for tests and full reporting of outcomes
☐	☒	Estimates of effect sizes (e.g. Cohen's *d*, Pearson's *r*), indicating how they were calculated

Our web collection on statistics for biologists contains articles on many of the points above.

Software and code

Policy information about availability of computer code

Data collection	LIQUID (https://github.com/PNNL-Comp-Mass-Spec/LIQUID)<br>MZmine 2 (http://mzmine.github.io)<br>ZEN (https://www.zeiss.com)<br>AxioVision (https://www.zeiss.com)<br>BD CellQuest Pro (http://www.bdbiosciences.com)
Data analysis	Prism v8.0 (https://www.graphpad.com)<br>MATLAB vR2016b (https://www.mathworks.com)<br>rMd-PAV (https://www.biopilot.org/docs/Software/RMD.php)<br>Yellowbrick (https://github.com/DistrictDataLabs/yellowbrick)<br>Fiji (https://imagej.net/Fiji)<br>Openlab (http://www.perkinelmer.com)<br>Cytoscape (https://cytoscape.org)<br>MetScape (http://metscape.med.umich.edu)<br>MetDisease (http://metdisease.ncibi.org)<br>JACoP (https://imagej.nih.gov/ij/plugins/track/jacop.html)<br>FlowJo (https://www.flowjo.com)<br>Imaris 9.0 (https://www.bitplane.com)

For manuscripts utilizing custom algorithms or software that are central to the research but not yet described in published literature, software must be made available to editors and reviewers. We strongly encourage code deposition in a community repository (e.g. GitHub). See the Nature Research guidelines for submitting code & software for further information.

Data

Policy information about availability of data

All manuscripts must include a data availability statement. This statement should provide the following information, where applicable:

- Accession codes, unique identifiers, or web links for publicly available datasets
- A list of figures that have associated raw data
- A description of any restrictions on data availability

All mass spectrometry datasets generated during this study have been deposited at the Mass Spectrometry Interactive Virtual Environment (MassIVE) at the University of California at San Diego, (https://massive.ucsd.edu/ProteoSAFe/static/massive.jsp), under the ID code MSV000085584. All other data are available from the corresponding author upon request.

Field-specific reporting

Please select the one below that is the best fit for your research. If you are not sure, read the appropriate sections before making your selection.

[x] Life sciences [ ] Behavioural & social sciences [ ] Ecological, evolutionary & environmental sciences

For a reference copy of the document with all sections, see nature.com/documents/nr-reporting-summary-flat.pdf

Life sciences study design

All studies must disclose on these points even when the disclosure is negative.

Sample size	Experiments were generally performed in triplicate, as is standard. For colocalization studies, the number of individual cells for each analysis was pre-determined.
Data exclusions	A single lipidomics sample (ZIKV 24 hpi) was identified as an outlier via rMd-PAV and Pearson correlation between all samples, and was excluded from further analysis as detail in the Methods. Criteria for exclusion was predetermined.
Replication	Experiments were successfully repeated as stated in the relevant figure legends.
Randomization	This study did not include experiments for which randomization was applicable.
Blinding	This study did not include experiments for which blinding was applicable.

Reporting for specific materials, systems and methods

We require information from authors about some types of materials, experimental systems and methods used in many studies. Here, indicate whether each material, system or method listed is relevant to your study. If you are not sure if a list item applies to your research, read the appropriate section before selecting a response.

Materials & experimental systems

n/a	Involved in the study	
[ ]	[x]	Antibodies
[ ]	[x]	Eukaryotic cell lines
[x]	[ ]	Palaeontology and archaeology
[x]	[ ]	Animals and other organisms
[x]	[ ]	Human research participants
[x]	[ ]	Clinical data
[x]	[ ]	Dual use research of concern

Methods

n/a	Involved in the study	
[x]	[ ]	ChIP-seq
[ ]	[x]	Flow cytometry
[x]	[ ]	MRI-based neuroimaging

Antibodies

Antibodies used	Anti-ZIKV NS4B (GeneTex, Cat# GTX133311, Lot# 42508); anti-Calnexin Alexa Fluor 647 (Thermo Fisher, Cat#MA3-027, Clone AF18, Lot# UE286770); anti-TGN46 Alexa Fluor 647 (Novus Biologicals, Cat# NBP1-49643, Lot# A-082619-AF647); anti-Ceramide (Sigma Aldrich, Cat# C8104, Clone MID 15B4, Lot# SLBM6077V); rabbit anti-Flavivirus E (Novus Biologicals, Cat# NBP2-52666, Clone D1-4G2-4-15 (4G2), Lot# T1904A15); mouse anti-Flavivirus E (Novus Biologicals, Cat# NBP2-52709, Clone D1-4G2-4-15 (4G2)); anti-!-Actin HRP (Cell Signaling, Cat# 5125, Clone 13E5, Lot# 6); anti-Mouse Alexa Fluor 555 (Thermo Fisher, Cat# A21422; Lot# 1480471); anti-Rabbit Alexa Fluor 555 (Thermo Fisher, Cat# A21428; Lot# 1937183); anti-Rabbit Alexa Fluor 488 (Cat# A11034; Lot# 1971418); anti-Mouse HRP (Cell Signaling, Cat# 7076, Lot# 32).
Validation	All antibodies were validated by their respective manufacturers for the applications used in this manuscript.

Eukaryotic cell lines

Policy information about cell lines

Cell line source(s)	Immortalized cell lines were obtained from the Oregon Health & Science University Vaccine and Gene Therapy Institute and the Whitehead Institute for Biomedical Research. Human neural progenitor cells were purchased from StemCell Technologies.
Authentication	Immortalized cell lines were not authenticated. Neural progenitor cells were authenticated by the manufacturer.
Mycoplasma contamination	All cell lines tested negative for mycoplasma contamination.
Commonly misidentified lines (See ICLAC register)	No commonly misidentified cell lines were used.

Flow Cytometry

Plots

Confirm that:

- [x] The axis labels state the marker and fluorochrome used (e.g. CD4-FITC).
- [x] The axis scales are clearly visible. Include numbers along axes only for bottom left plot of group (a 'group' is an analysis of identical markers).
- [x] All plots are contour plots with outliers or pseudocolor plots.
- [x] A numerical value for number of cells or percentage (with statistics) is provided.

Methodology

Sample preparation	Details for sample preparation included in Methods. All cells used were Huh 7. Cells were scraped from dishes, washed with PBS, live-dead stained with Zombie Violet, stained with lysenin-GFP, washed in 3%FCS, fixed with 4% PFA, permeabilized and blocked with 3% FCS in 0.1% Triton x-100 solution, then internally stained with anti-ceramide antibody and anti-mouse Alexa Fluor 561 secondary antibody. Finally, cells were washed in FACS buffer (3% FCS, 1% EDTA in PBS) before running.
Instrument	BO FACSymphony
Software	CellQuest for data collection, FlowJo for data analysis
Cell population abundance	All cells used were Huh7 cells, grown and seeded equally in cell culture for use in the experiment
Gating strategy	SSC-Area by FSC-Area were used to find normal cells as the first gate. Next SSC-Height by FSC-Area was used to select for singlet cells. Antibody-staining was determined in comparison with unstained controls, though no gating was used.

- [x] Tick this box to confirm that a figure exemplifying the gating strategy is provided in the Supplementary Information.
