# Supplementary material for: A global lipid map defines a network essential for Zika virus replication
Source: Nat Commun. 2020 Jul 21;11:3652. doi: 10.1038/s41467-020-17433-9 (PMC7374707; doi:10.1038/s41467-020-17433-9)
Supplement: Supplementary file 4 — Description of Additional Supplementary Files [file 41467_2020_17433_MOESM4_ESM.pdf]

Description of Additional Supplementary Files

File Name: Supplementary Data 1
Description: **Profile of changes in the global lipidome during ZIKV infection.**
Identified lipids in mock and infected Huh7 cells. The ionization mode, associated adduct, m/z, retention time (RT), p-values, and log2 fold change is provided for each lipid. The flag is an indication of significance (see notes section). Lipid annotations annotated with 'A' and 'B' refer to lipid structural isomers. If the exact acyl chains are not known, the lipid chains will be collapsed into total number of carbons and double bond (e.g., TG(44:2)).

File Name: Supplementary Data 2
Description: **Profile of changes in the global lipidome during ZIKV NS4B expression.**
Identified lipids in HEK293T cells transfected with ZIKV NS4B or an empty vector. The ionization mode, associated adduct, m/z, retention time (RT), p-values, and log2 fold change (FC) is provided for each lipid. The flag is an indication of significance (see notes section). Lipid annotations annotated with 'A' and 'B' refer to lipid structural isomers. If the exact acyl chains are not known, the lipid chains will be collapsed into total number of carbons and double bond (e.g., TG(44:2)).

File Name: Supplementary Data 3
Description: **Profile of changes in the global lipidome during treatment with myriocin or FB1.**
Identified lipids in Huh7 cells treated for three days with myriocin or FB1. The ionization mode, associated adduct, m/z, retention time (RT), p-values, and log2 fold change is provided for each lipid. The flag is an indication of significance (see notes section). Lipid annotations annotated with 'A' and 'B' refer to lipid structural isomers. If the exact acyl chains are not known, the lipid chains will be collapsed into total number of carbons and double bond (e.g., TG(44:2)).

File Name: Supplementary Data 4
Description: **Calculation and statistical analysis of SM-Cer pair ratios.**
Ratios of normalized intensities for each possible paired combination of SM and Cer species. The variation of each ratio across timepoints (24 and 48 hpi) and infection status (ZIKV or Mock) was assessed for statistical significance by ANOVA, and for biological significance by principal component analysis (PCA) and recursive feature elimination with cross-validation (RFECV).

File Name: Supplementary Data 5
Description: **Nodes of metabolic network connecting lipid subclasses.**
Contains identifying information for the gene and compound nodes of the global lipid metabolic network constructed by the Metscape plugin for Cytoscape. All identifiers are associated in Metscape.
